# Supplementary material for: Characterising motor and cognitive contributions of cortical beta oscillations and their modulation with rTMS
Source: Neuroimage. 2026 May 1;331:121893. doi: 10.1016/j.neuroimage.2026.121893 (PMC13328065; doi:10.1016/j.neuroimage.2026.121893)
Supplement: Supplementary file 1 [file mmc1.docx]

**Supplementary Table 1.** Comparisons on reaction times across different uncertainty and rTMS conditions using generalised linear mixed effect (GLME) modelling

| **Model 1**: RT ~ 1 + $k_{1}\mathrm{uncerID}$+ $k_{2}\mathrm{stimID}$ + $k_{inter}\mathrm{uncerID}*\mathrm{stimID}+$ 1\|subID + (-1 + uncerID\|subID) + (-1 + stimID\|subID) | | | | | | | |
| --- | --- | --- | --- | --- | --- | --- | --- |
| AIC | $k_{1}$ | $p_{1}$ | $k_{2}$ | $p_{2}$ | $k_{inter}$ | $p_{inter}$ | $R^{2}$ |
| -15611 | 0.0680 ± 0.0042 | **< 0.0001** | -0.0109 ± 0.0034 | **0.0016** | -0.0013 ± 0.0014 | 0.3353 | 0.4565 |
| -11683 | 0.0682 | **100%** | -0.0130 | **100%** | -0.0011 | 0 | 0.4660 |
| **Model 2**: RT ~ 1 + $k_{1}\mathrm{uncerID}$+ $k_{2}\mathrm{stimID}$ + $k_{inter}\mathrm{uncerID}*\mathrm{stimID}+$ 1\|subID + (-1 + uncerID\|subID) + (-1 + stimID\|subID), exclude stimID == 3 | | | | | | | |
| AIC | $k_{1}$ | $p_{1}$ | $k_{2}$ | $p_{2}$ | $k_{inter}$ | $p_{inter}$ | $R^{2}$ |
| -9639.5 | 0.0742 ± 0.0055 | **< 0.0001** | -0.0190 ± 0.0071 | **0.0070** | -0.0055 ± 0.0030 | 0.0621 | 0.4402 |
| -76551 | 0.0710 | **100%** | -0.0257 | **100%** | -0.0032 | 20% | 0.4559 |
| **Model 3**: RT ~ 1 + $k_{1}\mathrm{uncerID}$+ $k_{2}\mathrm{stimID}$ + $k_{inter}\mathrm{uncerID}*\mathrm{stimID}+$ 1\|subID + (-1 + uncerID\|subID) + (-1 + stimID\|subID), exclude stimID == 2 | | | | | | | |
| AIC | $k_{1}$ | $p_{1}$ | $k_{2}$ | $p_{2}$ | $k_{inter}$ | $p_{inter}$ | $R^{2}$ |
| -9660.5 | 0.0707 ± 0.0047 | **< 0.0001** | -0.0107 ± 0.0036 | **0.0028** | -0.0019 ± 0.0015 | 0.2016 | 0.4530 |
| -7612.3 | 0.0691 | **100%** | -0.0131 | **100%** | -0.0011 | 0 | 0.4621 |
| **Model 4**: RT ~ 1 + $k_{1}\mathrm{uncerID}$+ $k_{2}\mathrm{stimID}$ + $k_{inter}\mathrm{uncerID}*\mathrm{stimID}+$ 1\|subID + (-1 + uncerID\|subID) + (-1 + stimID\|subID), exclude stimID == 1 | | | | | | | |
| AIC | $k_{1}$ | $p_{1}$ | $k_{2}$ | $p_{2}$ | $k_{inter}$ | $p_{inter}$ | $R^{2}$ |
| -12104 | 0.0586 ± 0.0067 | **< 0.0001** | -0.0034 ± 0.0053 | 0.5242 | 0.0022 ± 0.0024 | 0.3649 | 0.4987 |
| -11342 | 0.0609 | **100%** | -0.0005 | 0% | 0.0011 | 0% | 0.4954 |

RT=reaction time; uncerID=uncertainty condition index, 1, 2, and 3 indicate no, low, and high uncertainty conditions, respectively; stimID=rTMD condition index, 1, 2, and 3 indicate no, regular, and irregular rTMS conditions, respectively; inter=interaction; subID=subject index; AIC=Akaike information criterion. In each model, an independent random slope(s) between the predictor(s) and the dependent variable as well as an independent random intercept (s) were included. For each GLME model, the parameters were estimated based on maximum likelihood using Laplace approximation, the AIC, estimate value with standard error of the coefficient (*k* ± *SE*), pre-corrected *p*-value (*p*), and proportion of variability in the response explained by the fitted model (𝑅^2^) were reported. P-values survive multiple comparison correction following FDR approach were highlighted. The bottom row of each model reports the results of a sensitivity analysis in which the model was re-run on a balanced subsample created by randomly down-sampling to the minimum number of trials per cell (500 repetitions). The table presents the mean values for AIC, K, and R², as well as the percentage of significant p-values.

**Supplementary Table 2.** Comparisons on bilateral beta modulation across different uncertainty conditions in the absence of rTMS using generalised linear mixed effect (GLME) modelling

| **Model 1**: betaPow ~ 1 + $k_{1}\mathrm{uncerID}$+ $k_{2}\mathrm{laterID}$ + $k_{inter}\mathrm{uncerID}*\mathrm{laterID}+$ 1\|subID + (-1 + uncerID\|subID), exclude stimID ≠ 1 | | | | | | | |  |
| --- | --- | --- | --- | --- | --- | --- | --- | --- |
| AIC | $k_{1}$ | $p_{1}$ | $k_{2}$ | $p_{2}$ | $k_{inter}$ | $p_{inter}$ | $R^{2}$ |  |
| 18524 | 0.3800 ± 0.2196 | 0.0836 | 0.3983 ± 0.3057 | 0.1927 | 0.0205 ± 0.1388 | 0.8825 | 0.0296 |  |
| **Interpret:** No interaction between uncertainty and laterality, therefore the model was updated by removing the interaction term. | | | | | | | |  |
| **Model 2**: betaPow ~ 1 + $k_{1}\mathrm{uncerID}$+ $k_{2}\mathrm{laterID}$ + 1\|subID + (-1 + uncerID\|subID), exclude stimID ≠ 1 | | | | | | | |  |
| AIC | $k_{1}$ | $p_{1}$ | $k_{2}$ | $p_{2}$ |  |  | $R^{2}$ |  |
| 18522 | 0.4108 ± 0.0696 | **3.9747 × 10^-9^** | 0.4403 ± 0.1117 | **8.2834 × 10^-5^** |  |  | 0.0298 |  |
| **Interpret:** Significant main effects of uncertainty and laterality. | | | | | | | |  |
| **Model 3**: betaPow ~ 1 + $k_{1}\mathrm{uncerID}$ + 1\|subID, exclude stimID ≠ 1 \| laterID == 2 \| uncerID == 3 | | | | | | | |  |
| AIC | $k_{1}$ | $p_{1}$ |  |  |  |  | $R^{2}$ |  |
| 6043.9 | 0.2796 ± 0.1972 | 0.1565 |  |  |  |  | 0.0527 |  |
| **Model 4**: betaPow ~ 1 + $k_{1}\mathrm{uncerID}$ + 1\|subID, exclude stimID ≠ 1 \| laterID == 2 \| uncerID == 2 | | | | | | | |  |
| AIC | $k_{1}$ | $p_{1}$ |  |  |  |  | $R^{2}$ |  |
| 6079.6 | 0.4053 ± 0.1006 | **6.0167 × 10^-5^** |  |  |  |  | 0.0266 |  |
| **Model 5**: betaPow ~ 1 + $k_{1}\mathrm{uncerID}$ + 1\|subID, exclude stimID ≠ 1 \| laterID == 2 \| uncerID == 1 | | | | | | | |  |
| AIC | $k_{1}$ | $p_{1}$ |  |  |  |  | $R^{2}$ |  |
| 6537.8 | 0.4975 ± 0.1927 | **0.0099** |  |  |  |  | 0.0171 |  |
| **Interpret:** Pairwise comparisons showed significant differences between no and high, and between low and high uncertainty conditions on left hemisphere. | | | | | | | |  |
| **Model 6**: betaPow ~ 1 + $k_{1}\mathrm{uncerID}$ + 1\|subID, exclude stimID ≠ 1 \| laterID == 1 \| uncerID == 3 | | | | | | | |  |
| AIC | $k_{1}$ | $p_{1}$ |  |  |  |  | $R^{2}$ |  |
| 6012.3 | 0.3342 ± 0.1950 | 0.0869 |  |  |  |  | 0.0306 |  |
| **Model 7**: betaPow ~ 1 + $k_{1}\mathrm{uncerID}$ + 1\|subID, exclude stimID ≠ 1 \| laterID == 1 \| uncerID == 2 | | | | | | | |  |
| AIC | $k_{1}$ | $p_{1}$ |  |  |  |  | $R^{2}$ |  |
| 5956.3 | 0.4149 ± 0.0953 | **1.4618 × 10^-5^** |  |  |  |  | 0.0328 |  |
| **Model 8**: betaPow ~ 1 + $k_{1}\mathrm{uncerID}$ + 1\|subID, exclude stimID ≠ 1 \| laterID == 1 \| uncerID == 1 | | | | | | | |  |
| AIC | $k_{1}$ | $p_{1}$ |  |  |  |  | $R^{2}$ |  |
| 6419.1 | 0.5120 ± 0.1851 | **0.0058** |  |  |  |  | 0.0054 |  |
| **Interpret:** Pairwise comparisons showed significant differences between no and high, and between low and high uncertainty conditions on right hemisphere. | | | | | | | | |
| **Model 9**: $\mathrm{uncerID}$ ~ 1 + $k_{1}\mathrm{betaPow}$ + $k_{2}\mathrm{alphaPow}$ + 1\|subID, exclude stimID ≠ 1 \| laterID == 2, only consider power modulation on the left hemisphere. | | | | | | | |  |
| AIC | $k_{1}$ | $p_{1}$ | $k_{2}$ | $p_{2}$ |  |  | $R^{2}$ |  |
| 4233.4 | 0.0134 ± 0.0059 | **0.0239** | 0.0092 ± 0.0046 | **0.0471** |  |  | 0.0083 |  |
| **Model 10**: $\mathrm{uncerID}$ ~ 1 + $k_{1}\mathrm{betaPow}$ + $k_{2}\mathrm{alphaPow}$ + 1\|subID, exclude stimID ≠ 1 \| laterID == 1, only consider power modulation on the right hemisphere. | | | | | | | |  |
| AIC | $k_{1}$ | $p_{1}$ | $k_{2}$ | $p_{2}$ |  |  | $R^{2}$ |  |
| 4222 | 0.0200 ± 0.0065 | **0.0021** | 0.0125 ± 0.0046 | **0.0062** |  |  | 0.0151 |  |
| **Compare (Model 9, Model 10)** | | | | | | | |  |
| LRStat | deltaDF | pValue |  |  |  |  |  |  |
| 11.377 | 0 | **<0.0001** |  |  |  |  |  |  |
| **Interpret:** Modulation of beta and alpha power in both hemispheres can predict the level of uncertainty, with greater predictive accuracy observed when using modulation from the right hemisphere. | | | | | | | | |

betaPow=normalised beta power after the uncertainty cue; uncerID=uncertainty condition index, 1, 2, and 3 indicate no, low, and high uncertainty conditions, respectively; laterID=laterality condition index, 1 and 2 indicate the left and right hemispheres, respectively; inter=interaction; subID=subject index; stimID=rTMD condition index, 1, 2, and 3 indicate no, regular, and irregular rTMS conditions, respectively; AIC=Akaike information criterion; alphaPow=normalised alpha power after the uncertainty cue; LRStat= Likelihood Ratio Statistic; deltaDF=The difference in degrees of freedom between the models; pValue= statistical p-value indicating whether the improvement in fit is statistically significant or not. Each model included an independent random intercept to count for variability across participants. The inclusion of random slop(s) between the predictor(s) and the dependent variable was determined through model comparison. For each GLME model, the parameters were estimated based on maximum likelihood using Laplace approximation, the AIC, estimate value with standard error of the coefficient (*k* ± *SE*), pre-corrected *p*-value (*p*), and proportion of variability in the response explained by the fitted model (𝑅^2^) were reported. P-values survive multiple comparison correction following FDR approach were highlighted.

**Supplementary Table 3.** Generalized linear mixed-effects (GLME) modelling revealed no significant association between reaction time and the timing of rTMS pulses in the rTMS conditions.

| **Model 1**: RT ~ 1 + $k_{1}\mathrm{uncerID}$+ $k_{2}\mathrm{lag}$ + $k_{inter}\mathrm{uncerID}*\mathrm{lag}+$ 1\|subID + (-1 + uncerID\|subID), exclude stimID == 1 | | | | | | | |
| --- | --- | --- | --- | --- | --- | --- | --- |
| AIC | $k_{1}$ | $p_{1}$ | $k_{2}$ | $p_{2}$ | $k_{inter}$ | $p_{inter}$ | $R^{2}$ |
| -12507 | 0.1026 ± 0.0201 | **3.5545 × 10^-7^** | 0.0060 ± 0.0458 | 0.8959 | -0.0510 ± 0.0205 | **0.0129** | 0.5430 |
| **Interpret:** Significant interaction between uncertainty and lag, therefore the model was updated by considering each individual uncertainty level. | | | | | | | |
| **Model 2**: RT ~ 1 + $k_{1}\mathrm{lag}$ $+$ 1\|subID, exclude stimID == 1 \| uncerID ≠ 1 | | | | | | | |
| AIC | $k_{1}$ | $p_{1}$ |  |  |  |  | $R^{2}$ |
| -3498.8 | 0.0182 ± 0.0434 | 0.6756 |  |  |  |  | 0. 1965 |
| **Model 3**: RT ~ 1 + $k_{1}\mathrm{lag}$ $+$ 1\|subID, exclude stimID == 1 \| uncerID ≠ 2 | | | | | | | |
| AIC | $k_{1}$ | $p_{1}$ |  |  |  |  | $R^{2}$ |
| -4387.2 | -0.1256 ± 0.0609 | 0.0444 |  |  |  |  | 0. 3216 |
| **Model 4**: RT ~ 1 + $k_{1}\mathrm{lag}$ $+$ 1\|subID, exclude stimID == 1 \| uncerID ≠ 3 | | | | | | | |
| AIC | $k_{1}$ | $p_{1}$ |  |  |  |  | $R^{2}$ |
| -4599 | -0.0198 ± 0.0531 | 0.7092 |  |  |  |  | 0. 3417 |
| **Interpret:** There is no significant association between lag and reaction time in rTMS conditions. | | | | | | | |

RT=reaction time; uncerID=uncertainty condition index, 1, 2, and 3 indicate no, low, and high uncertainty conditions, respectively; lag=time lag/interval between the first TMS pulse and the go cue; inter=interaction; subID=subject index; stimID=rTMD condition index, 1, 2, and 3 indicate no, regular, and irregular rTMS conditions, respectively; AIC=Akaike information criterion. In each model, an independent random slope(s) between the predictor(s) and the dependent variable as well as an independent random intercept (s) were included. For each GLME model, the parameters were estimated based on maximum likelihood using Laplace approximation, the AIC, estimate value with standard error of the coefficient (*k* ± *SE*), pre-corrected *p*-value (*p*), and proportion of variability in the response explained by the fitted model (𝑅^2^) were reported. P-values survive multiple comparison correction following FDR approach were highlighted.

**Supplementary Table 4.** Generalized linear mixed-effects (GLME) modelling revealed no significant associations between reaction time or uncertainty level and mean beta power around movement onset.

| **Model 1**: RT ~ 1 + $k_{1}\mathrm{uncerID}$+ $k_{2}\mathrm{beta}_{onset}$ + $k_{inter}\mathrm{uncerID}*\mathrm{beta}_{onset}+$ 1\|subID + (-1 + uncerID\|subID), exclude stimID ≠ 1 | | | | | | | |
| --- | --- | --- | --- | --- | --- | --- | --- |
| AIC | $k_{1}$ | $p_{1}$ | $k_{2}$ | $p_{2}$ | $k_{inter}$ | $p_{inter}$ | $R^{2}$ |
| -3744.7 | 0.0720 ± 0.0186 | **0.0001** | 0.0004 ± 0.0016 | 0.7790 | -0.0001 ± 0.0007 | 0.8408 | 0.3902 |
|  |  |  |  |  |  |  |  |
| **Interpret:** No significant association between reaction time and mean beta power around movement onset. | | | | | | | |
| **Model 2**: uncerID ~ 1 + $k_{1}\mathrm{beta}_{onset}+$ 1\|subID, exclude stimID ≠ 1 | | | | | | | |
| AIC | $k_{1}$ | $p_{1}$ |  |  |  |  | $R^{2}$ |
| 4248.1 | -0.0010 ± 0.0051 | 0.8511 |  |  |  |  | 1.9986e-05 |
| **Interpret:** No significant association between uncertainty level and mean beta power around movement onset. | | | | | | | |

RT=reaction time; uncerID=uncertainty condition index, 1, 2, and 3 indicate no, low, and high uncertainty conditions, respectively; $\mathrm{beta}_{onset}$=mean beta power within a 200 ms window surrounding movement onset; subID=subject index; stimID=rTMD condition index, 1, 2, and 3 indicate no, regular, and irregular rTMS conditions, respectively; AIC=Akaike information criterion. In each model, an independent random slope(s) between the predictor(s) and the dependent variable as well as an independent random intercept (s) were included. For each GLME model, the parameters were estimated based on maximum likelihood using Laplace approximation, the AIC, estimate value with standard error of the coefficient (*k* ± *SE*), pre-corrected *p*-value (*p*), and proportion of variability in the response explained by the fitted model (𝑅^2^) were reported. P-values survive multiple comparison correction following FDR approach were highlighted.


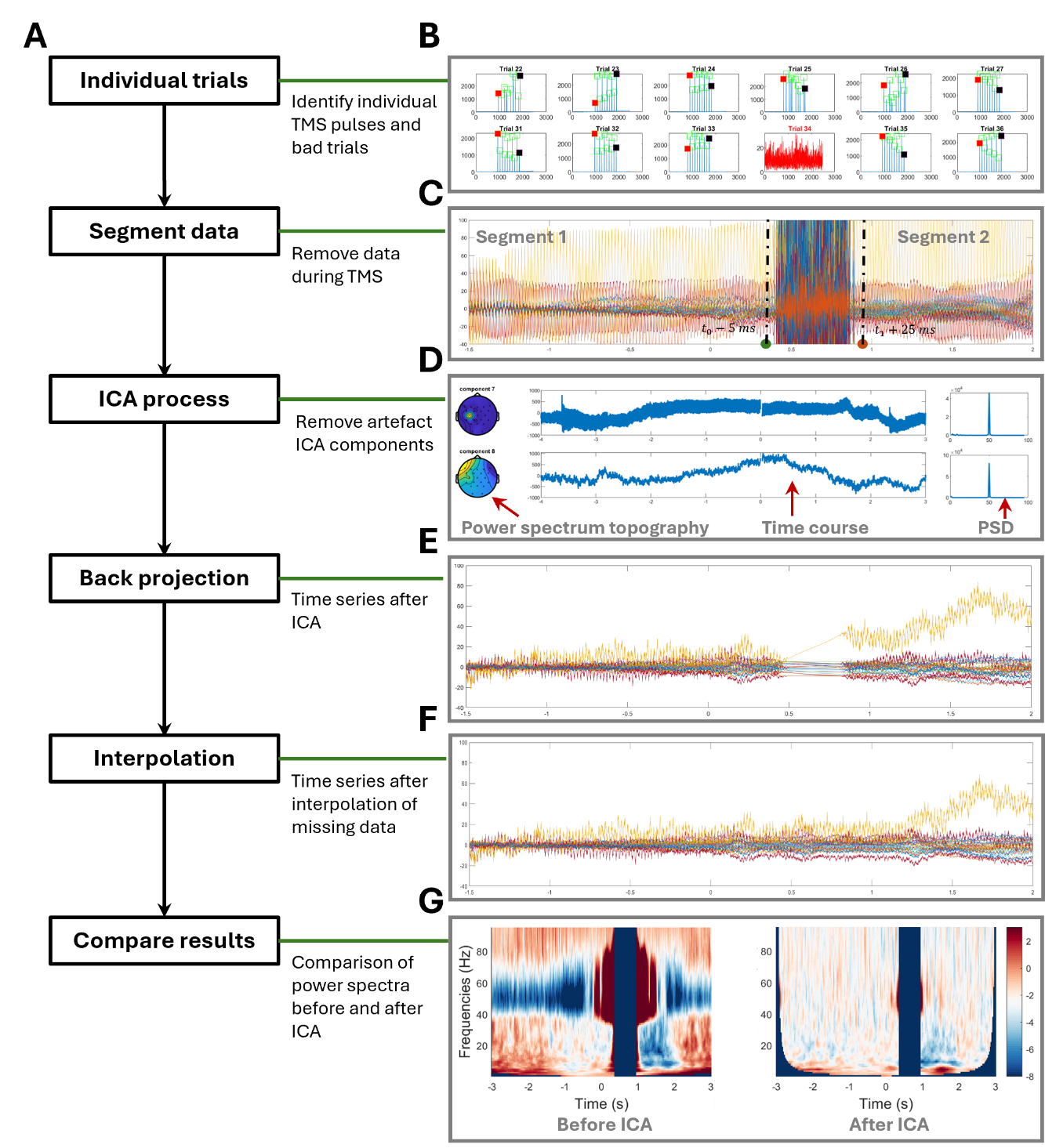


**Supplementary Fig. 1. EEG data preprocessing pipeline. (A)** Schematic of the preprocessing steps used in this study. **(B)** Identification of individual TMS pulses and exclusion of bad trials. **(C)** Removal of data from 5 ms before the first TMS pulse to 25 ms after the last TMS pulse. **(D)** Identification and removal of artefactual components using Independent Component Analysis. **(E)** Reconstruction of the time series from the cleaned components. **(F)** Interpolation of data segments missing during TMS pulses by mirroring the signal immediately before and after each pulse to ensure continuity. **(G)** Example comparison of power spectra before and after preprocessing.


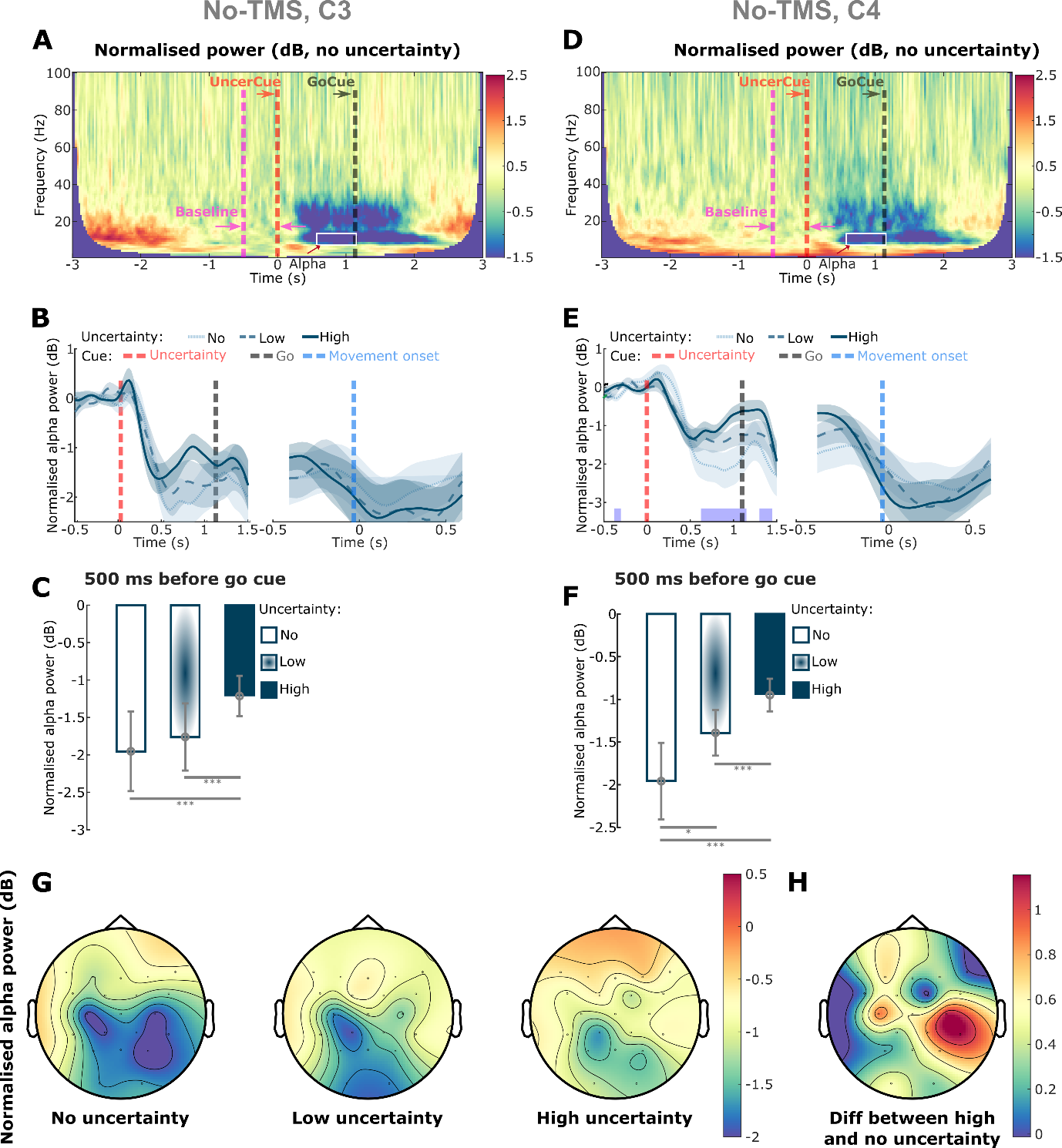


**Supplementary Fig. 2. Bilateral modulation of alpha by uncertainty. (A)** Group-averaged time-frequency power spectra from EEG channel C3 (left hemisphere), aligned to the onset of the ‘uncertainty’ cue (red dashed line), in the no-rTMS/no-uncertainty condition. Power spectra were normalised to a 500-millisecond pre-cue resting baseline for each trial. Alpha-band power (white box) showed modulations following the ‘uncertainty’ cue and prior to the ‘go’ cue (black dashed line). **(B)** Group-averaged time courses of alpha power, normalised to baseline, in different uncertainty conditions. Red, black, and blue dashed lines indicate the ‘uncertainty’, ‘go’, and ‘movement onset’ cues, respectively. **(C)** Comparison of alpha power modulation across different uncertainty conditions. Power was quantified as the average within 500-millisecond window preceding the ‘go’ cue, normalised (in dB) to the 500-millisecond pre-‘uncertainty’ cue baseline. Error bars represent the mean ± SEM across participants. **(D)-(F)** Same analyses as in (A)-(C), but for EEG channel C4 (right hemisphere). **(G)** Topographical map of alpha power modulation under conditions of no (left), low (middle), and high (right) uncertainty. Power was quantified as in (C) and (F). **(H)** Difference in alpha power modulation between high and no uncertainty conditions. *P < 0.05, **P < 0.01, ***P < 0.001. P-values in (C) and (F) were derived from generalised linear mixed-effects models applied to individual trials and corrected for multiple comparisons using FDR. Purple bars in (B) and (E) indicate significant differences between no- and high-uncertainty conditions (cluster-based permutation test).


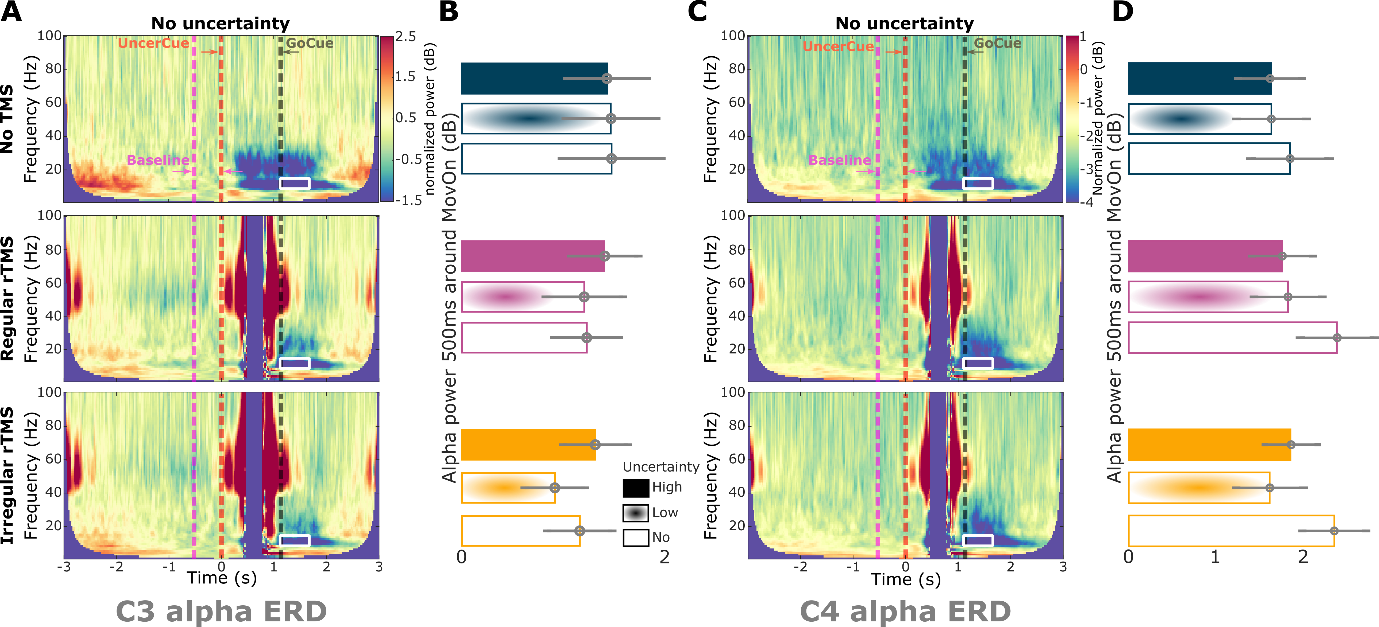


**Supplementary Fig. 3. Alpha ERD was not modulated with rTMS.** **(A)** Group-averaged time-frequency power spectra from EEG channel C3 (left hemisphere), aligned to the onset of the ‘uncertainty’ cue (red dashed line), shown for three conditions: no uncertainty and no TMS (up), regular rTMS (middle), and irregular rTMS (bottom). Power spectra were normalised to a 500-millisecond pre-cue resting baseline (pink dashed line) for each trial. Alpha-band activity (white box) showed modulation following movement onset (black dashed line). **(B)** Comparison of alpha ERD across different uncertainty and rTMS conditions. Power was quantified as the average within a 500-ms window preceding the ‘uncertainty’ cue and normalised (in dB) to the 500 ms window centered at movement onset. Error bars represent mean ± SEM across participants. **(C)-(D)** Same analyses as in (A)-(B), but for EEG channel C4 (right hemisphere). *P < 0.05. P-values in (B) and (D) were obtained using generalised linear mixed-effects models applied to individual trials and corrected for multiple comparisons using FDR.


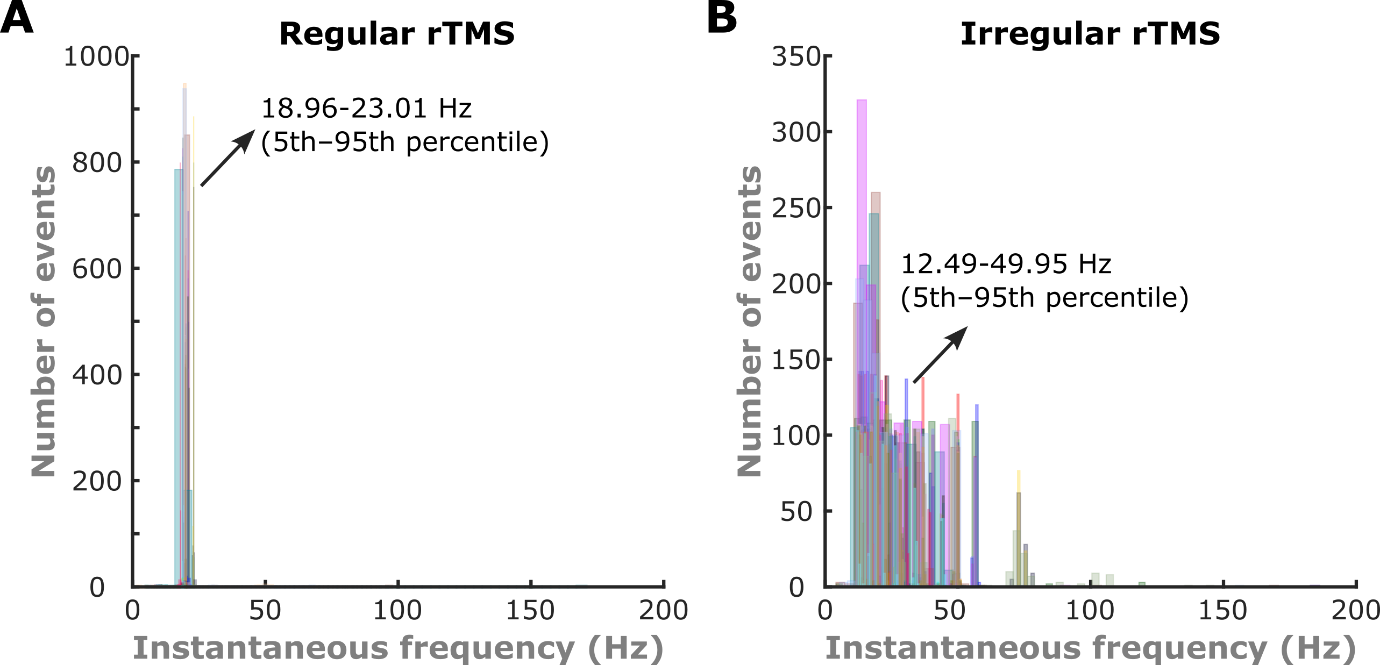


**Supplementary Fig. 4. Histogram of instantaneous TMS pulse frequencies in the regular and irregular rTMS conditions. (A)** In the regular rTMS condition, instantaneous frequencies ranged from 18.96 to 23.01 Hz (5th–95th percentile). **(B)** In the irregular rTMS condition, instantaneous frequencies ranged from 12.49 to 49.95 Hz (5th–95th percentile). Instantaneous frequency was computed by identifying the timing of each TMS pulse, calculating inter-pulse intervals, and converting these intervals to frequencies.


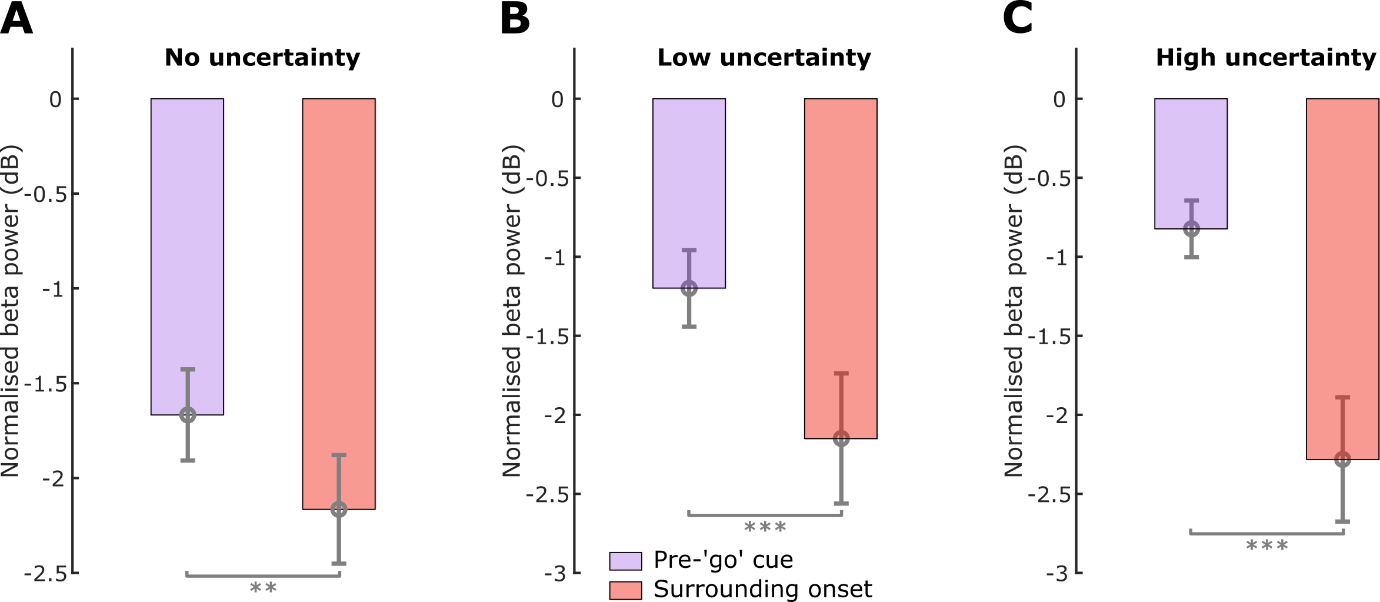


**Supplementary Fig. 5. Beta power is significantly reduced at movement onset compared to the pre-‘go’ cue period. (A)-(C)** Comparisons of mean beta power within the 500 ms preceding the ‘go’ cue and within a 200 ms window surrounding movement onset, shown separately for the no (A), low (B), and high (C) uncertainty conditions in the absence of rTMS.
